# Modelling zoonotic diseases in humans: comparison of methods for hantavirus in Sweden

**DOI:** 10.1186/1476-072X-11-39

**Published:** 2012-09-17

**Authors:** Caroline B Zeimes, Gert E Olsson, Clas Ahlm, Sophie O Vanwambeke

**Affiliations:** 1 Georges Lemaître Centre for Earth and Climate Research (TECLIM), Earth and Life Institute, Université catholique de Louvain (UCLouvain), Louvain, Belgium; 2 Department of Wildlife, Fish, and Environmental Studies, Swedish University of Agricultural Sciences, Umeå, Sweden; 3 Division of Infectious Diseases, Department of Clinical Microbiology, Umeå University Hospital, Umeå, Sweden

## Abstract

Because their distribution usually depends on the presence of more than one species, modelling zoonotic diseases in humans differs from modelling individual species distribution even though the data are similar in nature. Three approaches can be used to model spatial distributions recorded by points: based on presence/absence, presence/available or presence data. Here, we compared one or two of several existing methods for each of these approaches.

Human cases of hantavirus infection reported by place of infection between 1991 and 1998 in Sweden were used as a case study. Puumala virus (PUUV), the most common hantavirus in Europe, circulates among bank voles (*Myodes glareolus*). In northern Sweden, it causes nephropathia epidemica (NE) in humans, a mild form of hemorrhagic fever with renal syndrome.

Logistic binomial regression and boosted regression trees were used to model presence and absence data. Presence and available sites (where the disease may occur) were modelled using cross-validated logistic regression. Finally, the ecological niche model MaxEnt, based on presence-only data, was used.

In our study, logistic regression had the best predictive power, followed by boosted regression trees, MaxEnt and cross-validated logistic regression. It is also the most statistically reliable but requires absence data. The cross-validated method partly avoids the issue of absence data but requires fastidious calculations. MaxEnt accounts for non-linear responses but the estimators can be complex. The advantages and disadvantages of each method are reviewed.

## Introduction

### Modelling point records of presence of zoonotic disease

Zoonotic diseases are complex to model because pathogen presence in humans results from the interaction between humans, hosts, and the environment. In this way, species distribution modelling may be used, but interpretation of results may differ.

Many ecological and epidemiological spatial records are points. They relate to location-specific records of discrete units such as organisms or reported disease cases. A number of models allow investigating and predicting presence of organisms and pathogens based on a set of independent variables. These methods address in various ways the issue of confronting (or not) presences with absences. Recording a presence may be interpreted as a probabilistic function that depends on the abundance of the species/disease and on its detectability [1]. Absences, i.e. places where it is undoubted that the organism/pathogen is not present, may be recorded but are often a set of points randomly chosen through the study area. Absences may be interpreted in three ways [2]:

Environmental absences, related to unfavorable environmental and climatic conditions (not in potential or realized distribution),

Contingent absences, located in favorable areas (within the potential but not in the realized distribution) and,

Methodological absences, caused by a bias in the data collection.

If the ability to detect a species is constant across the study area (and differs from zero), then absences are reliable or associated to habitats where prevalence is low [1]. Absences of a zoonotic disease imply the absence of at least one of five elements: animal donor, vector, animal recipient, pathogen and an external environment allowing pathogen circulation [3]. Human records of zoonotic diseases can be assumed to approximate suitably the underlying zoonotic cycle if cases are well reported and human population distribution is relatively continuous. As animal data is challenging to collect, human data may offer a suitable alternative, especially when large areas are studied.

Models based on point data can be classified into three categories with regard to input data : presence and absence, presence and available, or presence-only. Here, representative methods of each approach were investigated and compared. First, binomial logistic regression, based on presence and absence data, was modeled. It fits a logistic curve between dependent variable and explanatory variables. Second, boosted regression trees (BRT) were tested. It is a decision tree where predictive performance is improved by boosting [4]. Third, cross-validated logistic regression (CV method), as introduced by Boyce et al. (2002), was computed. Points usually compared to presences may be better considered as undergoing a different intensity of use rather than being strict absences. The CV method considers available points instead of absence points [5]. Finally, an ecological niche model relying on presence-only data, specifically the MaxEnt model, was used. MaxEnt was chosen because it is frequently used and well documented [6,7]. Presence-only approaches use absences implicitly. The probabilities computed by the four models were mapped. The outputs of each model were compared using AUC and the kappa index. The presence and absence approach and presence-only approach have been compared [1,7-11] but here, in addition to comparing the predictive power or the goodness of fit, advantages and disadvantages are reviewed. Focus on modelling a zoonotic disease in humans implies the consideration of the preferences of multiple species.

### Case study: human hantavirus infections in northern Sweden

Human hantavirus infections were chosen as a zoonotic disease of public health importance in Europe, and a major rodent-borne disease [12]. In Sweden, Puumala hantavirus (PUUV), (*Bunyaviridae*)[13,14], is the most prevalent hantavirus and the only pathogenic one [15,16]. Its host is the bank vole (*Myodes glareolus*) [17]. In humans, PUUV causes nephropathia epidemica (NE) [18], a mild form of hemorrhagic fever with renal syndrome (HFRS) [19]. Transmission to humans may be direct by biting but is mainly indirect by breathing aerosolised urine and feces of infected voles [20]. Human infection often occurs during the cleaning of closed and un-aired buildings or while handling firewood [16,21,22]. At room temperature (and colder) and away from UV light, the virus remains infective for at least two weeks [20]. The number of recorded cases of HFRS in Europe (and in Sweden) has increased recently, which may be partly related to increasing surveillance and possibly to climatic factors [23-26]. In Scandinavia, the peak of NE occurs from November to December. Cases are however recorded year-round [27]. In Sweden, 90% of all NE cases notified are reported from the four northernmost counties [16].

Previous studies have showed that NE is linked to host abundance [16,25,28-30] and human risk activities (forestry, farming, wood cutting, construction work, camping, cleaning and/or redecorating building with rodents’ access …) [16,31]. Virus prevalence and transmission depend on local environmental, anthropogenic, genetic, behavioral and/or physiological factors [32]. Here we focused on environmental factors related to bank vole habitat, ex vivo virus survival and human presence, that influence the spatial distribution of disease [33].

## Materials and methods

### Data sources

**Figure 1:**
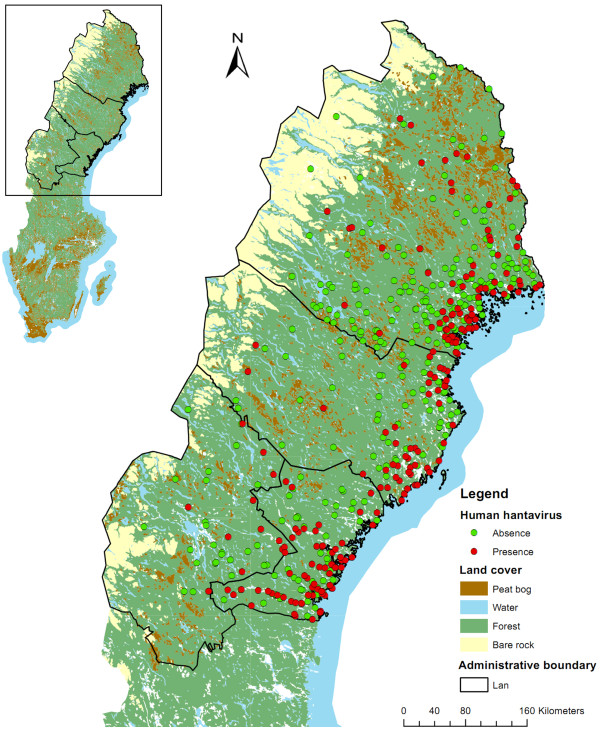
Human hantavirus infections in Sweden.

The study area covers the distribution range of hantavirus in Sweden (Figure 1) [34]. NE has been a notifiable disease in Sweden since 1989. In the present study, cases of NE recorded between 1991 and 1998 were used. Detailed locations of alleged sites of human PUUV exposure were acquired by mail and telephone survey.

During this period and in the region, a total of 1,724 cases of NE were notified, and 1,305 persons (76%) responded to the survey. Of these, 862 were confident about the time and location of PUUV exposure but only 217 could provide information detailed enough to link them to such an exact location as an estate. Data are reported by centroid of the land holding where the infection was acquired. Of the 217 cases recorded, some occurred in the same location. Only one record was kept for each location, leaving 212 presence points. 300 isolated dwellings were selected at random from the Lantmäteriet database (Swedish mapping, cadastral and land registration authority). They were used as absence points in the logistic regression and boosted regression trees and as available points in the CV model (Figure 1).

Three groups of environmental influences on the distribution of NE were explored (Table 1), relating to bank vole habitat, ex vivo virus survival, and human presence and exposure. In northern Sweden, the primary habitat of bank voles is mature and moist coniferous forest. Spruce forests are preferred over pine forests as they provide more food and shelter [35]. Forest data were extracted from the SLU Skogskarta (Sveriges lantbruksuniversitet: http://skogskarta.slu.se). The area of forests, the mean volume of spruce and the mean volume of pine were calculated in a radius of three kilometer around the infection place. Connectivity and contiguity of forests are important for the transmission of virus among bank voles on population level [36,37]. Due to their extensive coverage in the area, forests are all connected but locally, forest coverage and configuration vary. Landscape structure indices in a radius of three kilometers around the infection place were computed (number of forest patches, average shape index of forest, distance of the furthest cell from forests (using shortest path) , mean contiguity index of forest and mean Euclidian nearest-neighbor distance between patches of forests). As bank vole habitat is often related to peat bogs [16], the area of peat bogs in a three-kilometers radius was also calculated, based on the land cover data from Lantmäteriet.

**Table 1:** Independent variables and hypothesized relationships with the abundance of bank voles, the ex vivo virus survival and the human presence

**Variable in logistic model, BRT and CV model**	**Variable in MaxEnt model**	**Abundance of bank voles**	**Ex vivo virus survival**	**Human presence**	**Source**
Area of forests in a 3-km radius around the dwelling (m^2^)	Forests	x			SLU Skogskarta
*Mean volume of spruce per hectare in a 3-km radius around the dwelling (m^3^/ha)	Volume of spruce	x			SLU Skogskarta
Mean volume of pines per hectare in a 3-km radius around the dwelling (m^3^/ha)	Volume of pine	x			SLU Skogskarta
*Maximum distance to forests in a 3-km radius around the dwelling (m)		x			SLU Skogskarta
Number of patches of forests 3-km radius		x			SLU Skogskarta
Mean shape index of forests 3-km radius		x			SLU Skogskarta
Mean contiguity index of forests in a 3-km radius		x			SLU Skogskarta
Mean Euclidian nearest-neighbor distance between patches of forests in a 3-km radius (m)		x			SLU Skogskarta
*Area of peat bogs in a 3-km radius around the dwelling (m^2^)	Peat bogs	x			SVK
Mean snow depth between 1991 and 1998 (cm)	Snow depth	x	x		SMHI
Average duration of the snow when it is present for at least 10 days (days)	Snow period	x	x		SMHI
Majority of grain size of the soil (1 = coarse, 2 = medium, 3 = fine) in a 3-km radius	Soil grain size		x		SGU
*Elevation (m)	Elevation	x	x	x	Aster GDEM
*Distance to the sea coast (m)	Distance to the sea	x	x	x	SVK
*Population density (inhabitant/km^2^)	Population density			x	Gridded population of the world
Total length of public roads in a 3-km radius (m)	Roads			x	SVK
*Distance to holiday homes (m)	Holiday homes			x	Statistiska Centralbyran
Total length of the water ways in a 3-km radius (m)	Water ways			x	Swedish Places

* Data log-transformed.

Ex vivo virus survival depends on humidity and temperature [20]. Soil grain size was used as a proxy for soil humidity: soils with thinner particles will retain more moisture and allow better virus persistence [38,39]. Data on soil grain size were extracted from the Geological Survey of Sweden (SGU) and classified into coarse, medium and fine particles. A thick snow cover provides high levels of humidity, cold temperature and protection against UV light therefore contributing to better ex vivo virus survival [20]. Snow also affects abundance of bank voles by providing food and shelter preserved against harsh weather and predators [40-43]. Snow depth and average snow duration (when only present for at least 10 days) were computed from interpolated weather station data of the Swedish Meteorological and Hydrological Institute.

Human density presence follows a double gradient: from South to North and from East to West. Population density (Gridded Population of the World from Center for International Earth Science Information Network (CIESIN)) was used to reflect the spatial distribution of humans. Distance to the sea and elevation follow also this gradient. Elevation data were extracted from the Aster GDEM elevation data (Global Digital Elevation Model, Earth Remote Sensing Data Analysis Center (ERSDAC)). These two variables may act as proxies for climate, soil composition and attractiveness of the landscape. Other variables were also chosen to reflect human presence by attractiveness and accessibility of the landscape: distance to the nearest holiday house, extracted from the Central Statistical Bureau data (Satistiska Centralbyran) and, length of water ways and roads in a three kilometer radius (Lantmäteriet).

Independent variables with non-normal distribution were log-transformed (volume of spruce, distance to forests, area of peat bogs, elevation, distance to sea coast, population density and distance to holiday homes). For logistic regression, BRT and CV model, some variables were expressed as a value in a radius around the infection place, allowing consideration of the landscape encountered around the place of infection. MaxEnt however only allows spatially continuous variables and cannot integrate these variables in a straightforward fashion. Data layers included in MaxEnt were: forests, volume of spruce, volume of pine, peat bogs, soil grain size, snow depth, snow period, elevation, distance to the sea, population density, roads, holiday houses and water ways.

### Models

#### Presence vs. absence: logistic regression

Estimators are calculated by maximum likelihood to maximize the probability of obtaining the observed sample [44,45]. The intercept determines the position of the logistic curve on the dependent variable [46]. The coefficient of independent variable is the rate of change of the logit function per unit of change of the variable [44]. This estimator shows how fast the curve will increase or decrease.

First, univariate analyses (only one explanatory variable) were carried out with each independent variable. After, variables were selected for the multiple model using a backward stepwise procedure in R (“stats” package). The Akaike Information Criterions (AIC) was used to select the best model and the Variance Inflation Factor (VIF) (“car” package) was checked to avoid multicollinearity issues. Interactions between variables were tested but none was significant at the level of five percent. As quadratic terms decreased the goodness-of-fit of the model, they were not included.

#### Presence vs. absence: boosted regression trees

BRT combines decision trees with boosting to improve the performance (“gbm” package in R) [4]. In a regression tree, a branch leads to several internal nodes or to a terminal node. The path chosen at each internal node depends on the value of the explanatory variables. At a terminal node, a decision is made on presence or absence. Decision trees are built by recursive binary split: initial trees are enlarged by new binary split made on the previous trees. Boosting allows improving the optimization by adding new trees that reduce the most the loss in predictive performance. The procedure is forward and stage wise: after one step, a new tree is fitted on the residual of the previous tree and the new model, with new residuals, contains the previous and the new trees. BRT also include stochasticity defined by the bag fraction, the percentage of data randomly selected at each step. The default bag fraction is 0.5.

Three parameters must be defined: the learning rate, the tree complexity and the number of trees [4]. The learning rate is the contribution of each tree to the model. A low learning rate, which implies a larger number of trees, is advised. The tree complexity represents the number of nodes in a tree. A higher tree complexity implies thus a lower learning rate. Learning rate and tree complexity are chosen based on a visual analysis of graphs. Graphs represent, for a given tree complexity and at different learning rates, the loss of predictive performance (here, predictive deviance) according to the number of trees. A slower learning rate is generally preferable. The optimal number of trees is found when the predictive deviance is lowest.

The final tree is too big to be graphed but the contribution of each variable can be calculated and the effect of the variables (on the probabilities) graphed. Interactions are automatically modeled because the response of one variable depends of the previous responses of the other variables higher in the tree [4]. The relative strengths of interactions are reported and they can be plotted.

#### Presence vs. availability: cross-validated logistic regression

The CV method [5,47,48] is based on presence and available points. The presence of an organism depends on the presence of resources it uses. Each point has a different resource availability and hence of intensity of use (and not just the presence or absence of resources). The probability to find an organism in one place depends of its intensity of potential use. This method uses a classic logistic regression method, but the evaluation and use of the results focus on the computed probabilities and a cross-validation of the predicted probabilities. The variables selected by the stepwise procedure for the logistic regression model were used in this model. The data were divided five times, into five sub-samples. Five logistic regressions were calculated using each time a different combination of four sub-samples. The fifth sub-samples, not used for calibrating the model, were put together and used for validation. Estimators of the different regressions were averaged to produce the final model, which was applied to the validation sample to predict probabilities. The predicted probabilities calculated for the validation sample were clustered in 10 clusters of probabilities using quantiles. Here, validation requires calculating the utilization of resources for each cluster *U(x*_*i*_*)*:

(1) $$U\left(x_{i}\right)=\frac{w\left(x_{i}\right)A\left(x_{i}\right)}{\Sigma_{j}w\left(x_{i}\right)A\left(x_{i}\right)}$$

Where *w*(x_i_) is the mid-point probability of the cluster *i* and *A*(x_i_) is the area of cluster *i* (here, the number of observations in cluster i).

New predicted presences were calculated by multiplying *U(x*_*i*_*)* by the total number of observations for each class. These predicted presences can be compared with observed presences for each class:

1. Spearman coefficient (and *χ*^2^ test of goodness-of-fit) compared predicted and observed values. A high positive correlation is desired.

2. A linear regression of predicted cases (x) on observed cases (y) was modeled:

a. R^2^ was used to assess the predictive power.

b. The intercept was expected to be zero and the estimated regression coefficient was expected to be around one.

#### Presence only: MaxEnt model

MaxEnt is a model that maximizes entropy satisfying any constrain on the unknown distribution [49], and that minimizes the relative entropy between two probability densities defined in the covariate space, one estimated from the presence data and one from the landscape [50]. Unlike the two previous models, dependent variables are continuous spatial data layers with identical extent and resolution. Here, the extent was the four northern Swedish counties where NE is recorded and the resolution was one kilometer.

MaxEnt builds an occurrence model starting from a uniform distribution of probability for each cell of the raster [51]. Then, it improves the model iteratively until the gain becomes saturated. The gain is a likelihood statistic which maximizes the probability of presence according to the background data.

All dependent variables are used simultaneously. Collinear variables are usually not considered a problem because if a variable has a significant impact on probabilities, variables correlated to it will have little impact [50].

MaxEnt allows to account for sampling bias by including an additional layer representing the relative survey effort across the landscape. Population density layer was tested as sampling bias. MaxEnt also provides response curves showing the influence of a variable on the probability of presence. Jackknife analyses were used in order to evaluate the contribution of each variable to the model. Five-fold cross-validation was also used.

#### Comparison between models

Due to the limited size of the database, all data points were used for model training. No external validation was carried out, and internal indices were used to compare models.

As the logistic and CV models were built with the same variables and dataset, the Akaike’s Information Criteria (AIC) can be used. AIC is a measure of the goodness-of-fit of the model.

To evaluate the predictive power of the four models, the area under the curve (AUC), from a receiver operating characteristics (ROC) analysis, was calculated (“PresenceAbsence” package in R) [52,53]. The rate of true positives is plotted against the rate of false positives at all thresholds of classification into presence and absence. An AUC equals to 0.5 is a random distribution of predictions and an AUC equals to one, a perfect prediction.

Cohen’s kappa statistic, an index of agreement for positive and negative observations, was also calculated [54]. A kappa above 0.75 indicates an excellent agreement; between 0.4 and 0.75, a fair to good agreement and under 0.4, a poor agreement.

Probability maps were created for each model. MaxEnt provides a continuous probability map. For logistic regression, BRT and CV, predicted points probabilities were interpolated by kriging to obtain continuous maps.

False positives and false negatives were also mapped. Even if the CV model usually does not classify probabilities into presences and absences, a map was made for comparison. The probability threshold was chosen at the level where sensitivity (number of true positives divided by the sum of true positives and false negatives) equals specificity (number of true negatives divided by the sum of true negatives and false positives).

As inputs variables vary between methods and as MaxEnt AUC is calculated over the entire study area (presences and background), while the others only considered the set of points, AUCs and kappas were computed on an identical set of points and variables, in order to make an accurate comparison.

#### Partial analyses with variables related to bank voles, virus and humans

Partial logistic regressions, BRT and MaxEnt models were fitted using variables related to each element. In this manner, the relative importance of bank voles, virus and humans distributions on human infections distribution may be speculated.

## Results

### Logistic regression

Several variables were significant in the univariate analyses (p < 0.05):

with a positive sign: logarithm of mean volume of spruce, mean volume of pine, logarithm of distance to forests, logarithm of population density and,

with a negative sign: logarithm of area of bogs, snow depth, snow period, logarithm of elevation, logarithm of distance to sea, logarithm of distance to holiday home.

The multiple logistic model included six explanatory variables (Table 2). Forest contiguity and snow depth were retained in the stepwise procedure but were not significant (p > 0.05). The probability of presence increased with the area of forests, logarithm of distance to forests, contiguity and population density. It decreased with snow depth and logarithm of distance to sea.

**Table 2:** Models obtained by logistic regression and cross-validated logistic regression method

	**Estimate of logistic regression**	**Mean-estimate of CV method**
Intercept	−5.371**	−5.447
Area of forests	8.048*10^-8^***	8.133^-8^
Log (distance to forests)	1.665***	1.689
Contiguity	1.198	0.226
Snow depth	−0.016	−0.016
Log (distance to sea)	−0.470**	−0.471
Log (population density)	0.544*	0.109
AIC	629.74	792.77
AUC	0.972	0.721

* p-value < 0.05, ** p-value < 0.01 and *** p-value < 0.001.

**Figure 2:**
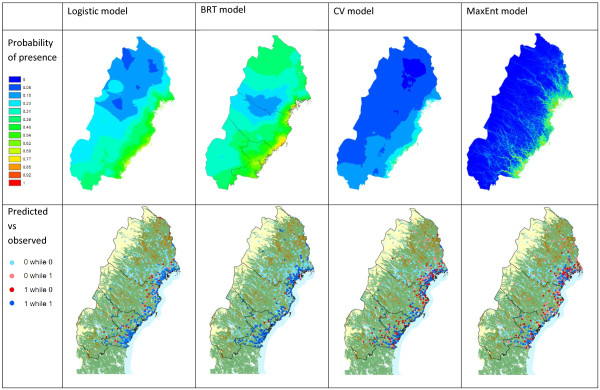
Comparison between results of logistic regression, boosted regression tree, cross-validated logistic regression and MaxEnt model.

With an AUC of 0.97 and a kappa index of 0.76, the logistic regression had a good predictive power and an excellent agreement. The probability of presence decreased from South to North and from East to West (Figure 2). False positives were found mostly in the East, where the model overestimated presence. False negatives were sparser.

### Boosted regression tree

A learning rate of 0.01 and a tree complexity of five were chosen based on visual analyses of graphs, giving an optimal number of trees of 350 for minimizing deviance.

Variables with the most important contributions were: area of forests (11.45%), distance to holiday homes (11.01%), distance to the sea (9.82%), elevation (8.81%) and mean volume of spruce (8.53%).

Interaction effects were the most important between the sum of roads and area of forests, the snow period and snow thickness, and the elevation and area of forests.

The AUC of 0.92 and kappa of 0.65 indicated a good model and a good agreement. Predicted probabilities generally increased from West to East, with an area of minimum probability in the center (Figure 2). The highest probabilities were found along the sea coast. No false absences were found and only 16 false positives.

### Cross-validated logistic regression

The estimated regression coefficients are found in Table 2. As these are averages, the significance degree was not known but no coefficient was close to zero. The Spearman correlation between observed and predicted values was significant (0.92; p-value = 0.0013). The linear regression between observed and predicted values had an adjusted-R^2^ of 0.84. Predicted values were slightly lower than observed values and not around one as expected (0.303; p-value < 0.001).

The AUC (0.72) indicated that the predictive power was satisfactory but the kappa (0.32) indicated a poor agreement. Probability of presence of disease decreased from South to North and from East to West. Most false positives were in the East (Figure 2).

### MaxEnt model

Results with and without the population density layer as sampling bias were similar. Jackknife analyses showed that elevation brought the highest gain when used in isolation from other variables. Roads decreased the gain most when omitted from the model.

The probability of presence increased with the volume of spruce and decreased with the volume of pines. Approximately after 60 m^3^/ha of pine, the probability of presence decreased, indicating that habitat is less favorable to bank voles. The two variables are probably complementary. When there are fewer pines, there are more spruces and vice-versa.

The AUC of MaxEnt model was good (0.91) but, when calculated on the same points than in logistic and CV model, it decreased to 0.66. Kappa was only calculated on the points and indicated poor agreement (0.19). The highest predicted probabilities were found near the sea coast, roads and water ways (Figure 2). There were many false positives in the East.

### Comparison between models

When accounting for the confidence interval, the estimators of logistic regression and CV method were similar, except for contiguity and population density (Table 2). AUC and AIC were best for logistic regression.

The linear pattern which appeared in MaxEnt was related to including spatially detailed data on roads and water ways (Figure 2). As the other models were based on points and then interpolated, such a linear pattern cannot appear, but may appear if probabilities were computed per pixel.

Based on the AUC, the logistic regression produced the best model. If a logistic regression is built with the same variables as MaxEnt and if the AUC of MaxEnt is calculated only on the original data points, both AUC were equal to 0.66 and ROC curve were similar, indicating similar goodness-of-fit.

The thresholds identified for classifying probabilities into presences and absences were 0.44 for logistic model, 0.42 for BRT, 0.17 for CV model and 0.49 for MaxEnt model. Except for BRT, all methods overestimated presence. Many false positives were near the sea coast.

### Partial analyses with variables related to bank voles, virus and humans

For logistic regressions and BRT, AUC were the best for models with variables related to bank vole habitat, followed by models related to virus and finally models related to humans (Table 3). Inversely, for the MaxEnt models, the best model was built on variables related to humans.

**Table 3:** AUC of partial models based on variables related to each element

	**Rodents**	**Virus**	**Humans**
Logistic regression	0.732	0.695	0.684
Boosted regression trees	0.886	0.8244	0.801
MaxEnt	0.891	0.893	0.922

## Discussion

Each method has advantages and disadvantages. Those pertaining to input data, ease of use, goodness of fit, predictive power and interpretation are reviewed here. A summary has been made in Table 4.

**Table 4:** Advantages and disadvantages of logistic regression, boosted regression trees, cross-validated logistic regression and Maxent model

	**Advantages**	**Disadvantages**
**Logistic regression**	-Best goodness-of-fit and predictive power	-Need of real absence points
	-Inclusion of variables reflecting the surrounding environment	
**BRT**	-Account for non-linearity of biological processes	-Need of real absence points
	-Modelling of interactions	-Impossible to see all three at one time
	-Inclusion of variables reflecting the surrounding environment	-Difficulty to extrapolate
**CV method**	-Available sites instead of absence sites	-Fastidious calculations
	-Inclusion of variables reflecting the surrounding environment	-Limited value compared to logistic regression
**Maxent model**	-Ease of use	-Complex estimators, difficulty to extrapolate
	-Spatially continuous results	-Need of spatially continuous data
	-Accounts for non-linearity of biological processes	-Limited by the coarsest resolution and the smallest extent of variables

### Input data

Logistic regression and BRT required absence data. Here, absences were identified from accurate data on dwellings but, these absence points may be unidentified cases or just an absence of human hantavirus transmission over the study period. Random locations would have been less appropriate as they would not consider human distribution. working on point data allowed the implementation of variables which reflect the surrounding environment such as the composition and configuration of the landscape. Zoonotic transmission indeed relates to factors extending beyond the place of record.

The CV method considered availability rather than absence, therefore avoiding the issue of unreported cases or absences related to the stochasticity of zoonotic disease transmission to humans. As data were points, independent variables reflecting the surrounding environment could also be included.

In MaxEnt, only presence records were required. Heavy constraints lied on the dependent variables (continuous raster maps of same resolution and geographical extent). Here, several variables at the landscape scale concerned landscape structure. This could not be operationalized as continuous variables in a comprehensive and straightforward fashion. On one hand, measures concerning the landscape surrounding infection sites could no longer be used. Continuous rasters could be constructed to represent the landscape variables, but loss of information is inevitable. On the other hand, the spatial pattern of the input variables, which played an important role for the final model was preserved. In partial analyses, the best MaxEnt fit used variables related to humans. The human population in Northern Sweden is highly structured along the sea coast and inland along roads and rivers. As the final model is calculated on each pixel, the linear pattern was more evident than in the others models.

### Ease of use

Logistic regression is widely available in statistical softwares and is easily implemented. The R package “gbm” used for BRT includes a user-friendly tutorial. The CV required fastidious calculations. MaxEnt, a free software with a graphical interface, is very user-friendly.

### Goodness-of-fit and predictive power

Generally, the logistic regression gave the best results. It had the best AUC and so, the best predictive power, followed by BRT and MaxEnt and, finally by CV model. It should be noted that BRT results were quite heavily influenced by the bag fraction. When the same independent variables were used in logistic and MaxEnt models, AUC and Kappa were comparable. So, the stepwise procedure and the input variables based on the surrounding environment allowed a better fit and predictive power.

False positives for a zoonotic disease can be interpreted as a poor prediction, a non-reported case, or the presence of the pathogen in the wild but its absence in humans. NE is generally under diagnosed, and many PUUV infected humans may go undetected. Indeed, up to seven in eight PUUV infected humans may go unrecognized with subclinical symptoms or symptoms mistaken [55]. In maps of predicted versus observed (Figure 2), CV and MaxEnt had more incorrect predictions, indicating a poorer prediction comparing to logistic regression and BRT. Models only based on presence were most likely to overestimate presence. However, false positives may give indications on the potential distribution, while the others approximate the realized distribution. The use of different sets of explanatory variables may also contribute to this, but tests using identical sets of predictors confirmed the results. All models overestimated presence near the sea coast, but BRT did the least.

### Interpretation

Logistic regression, BRT and CV models had higher flexibility for the inclusion of diverse variables. Variables with more straightforward biological interpretations and/or closer proxies could be added in the model. It could be argued that it is an attractive feature for explanatory models. In our case, landscape structure variables (e.g. relating to forest structure and arrangement with respect to human habitat) could be included. MaxEnt found powerful associations with altitude, a variable of little biological significance that proxies several other biologically relevant variables such as temperature, snow cover or population density. Use of MaxEnt may thus be less recommended to build explanatory models.

A major advantage of MaxEnt was the production of a spatially continuous result, allowing finer detail and more visually pleasing output and avoiding the necessity to interpolate results spatially. However, this may come at the price of many false positive pixels. It may still be useful for identifying further study sites. As the interpolated surfaces of the other methods are also uncertain, major risk areas could be first outlined by MaxEnt, then, in these areas, other models could be fitted to gain more detail.

MaxEnt and BRT facilitated the identification and interpretation of non-linear responses. Contributions and Jackknife analyses showed immediately interesting variables. Response curves showed the variation of probabilities in relation to the dependent variables. These curves brought a lot of information but could be quite complex to understand and a good understanding of the system was required. Moreover, in BRT, variables could be strongly correlated, meaning that curves were not reliable. Also, results may be difficult to generalize: in MaxEnt, non-linearity generated complex estimators and for the BRT, it was not possible to see all trees at one time.

Previous comparisons between presence/absence and presence-only methods have highlighted that logistic regression is more appropriate in some cases and MaxEnt are more appropriate in others. If absence data are available, logistic regression is better than MaxEnt to discriminate sites with high disease risk [56]. Penalized logistic regression, which avoids performance problems caused by overfitting, performs similarly to MaxEnt and has been found better than standard logistic regression [11]. Another study shows that MaxEnt is slightly better within the known distribution but logistic regression predicts better outside the data distribution [10].

Similar modeling approaches have been used for other hantaviruses. A study on Juquitiba hantavirus infections in humans in Brazil identified risk areas using MaxEnt [57]. The authors concluded that human data were limited for modeling the virus in host populations. A study in Argentine used reservoir host data and logistic regression to estimate risk areas for humans [58]. Another study on Andes hantavirus in Argentina comparing MaxEnt and logistic regression using rodent data and human infection data found good predictive powers for both methods in predicting rodent distribution, while MaxEnt performed less well on human data [59]. In partial analyses, the importance of bank vole distribution was highlighted in logistic regression and BRT. These models allowed including landscape structure variables that describe the rodent habitat in more detail. In our study, MaxEnt model indicated the importance of human distribution because of its spatial pattern. Modelling the spatial distribution of human hantavirus infections requires thus both environmental conditions and human variables.

Our four models were based on environmental conditions and tried to define the intersection of the spatial distributions of bank voles, humans and virus. Care is however needed when interpreting results, particularly differences between potential and realized distributions [8]. Even if all favorable conditions are present, the disease/species is not necessarily found. False positive results may be the result of non-transmission of the pathogen to humans even if it circulates in wild hosts. Moreover, as bank voles have a wide ecological niche, models are less accurate [60]. Modelling zoonotic diseases in humans is best done using human case data as host data often represents a broader distribution. Zoonoses involve several species as well as humans and their activities.

#### Further proposals

An option could be to use first BRT or MaxEnt, in order to delineate areas of high probabilities. Variables could then be sliced according to their response curve into several variables or transformed into categorical variable. This way, non-linear processes could be considered. If absences are available, they can be added in logistic regressions or, if not, in the CV method. Non-continuous landscape variables can then be added. The final purpose of the model, explicative or predictive, would also direct the choice, as would data availability and specificity of the system at hand.

#### Human hantavirus infections in Sweden

Disease cases were found at the intersection of the distribution of bank voles, humans and virus. Many factors must be taken into account. Distance to the sea, which was included in the logistic model, and elevation, which brought the highest gain in the MaxEnt model were proxies for different phenomena. These variables reflected a double gradient also represented by different explanatory variables. A milder climate is found near the coast and in the south, there were more spruces than pines, the soil was moister and human density was higher. Even if the correlation was not always strong, all variables were interconnected.

Variables included in the logistic model and BRT represented bank vole habitat and its connectivity, survival of the virus and human distribution. Distance to forests and contiguity were measures for connectivity of forest. The connectivity index must be taken with caution because it is not necessarily functional [61,62]. The habitat of bank voles and the virus-preserving snow cover were important. Other models of hantavirus infections around the world show the importance of land cover and climate [57-59]. In China, a MaxEnt study based on infected rodents highlighted the importance of land cover and elevation [63]. In the USA, a logistic regression model based on human infections showed the importance of elevation, climate and ecotone [64].

## Conclusion

Zoonoses, included the rodent-borne hantavirus, can be modelled with diverse methods. The methods presented here differed in what they permit and offer, each of which may be more important depending on the study objectives. Each method has advantages and disadvantages. A solution could be to combine the different methods

## Competing interests

The authors declare that they have no competing interests.

## Authors’ contributions

CZ and SV designed the study, interpreted the results and drafted the manuscript. CZ carried out the analysis. GO and CA collected the data and revised critically the manuscript. All authors read and approved the final manuscript.

## Aknowledgements

This study was funded by EU grants FP7-261504 EDENext and is catalogued by the EDENext Steering Committee as EDENext 054 (http://www.edenext.eu). The contents of this publication are the sole responsibility of the authors and don’t necessarily reflect the views of the European Commission. The initial work on data collection was supported by grants from the Centre for Environmental Research in Umeå, the County of Västerbotten, the Medical Faculty of Umeå University, and the County Councils of Northern Sweden. The authors thank N. Hartemink for reviewing.
